# Induction of renal senescence marker protein-30 (SMP30) expression by testosterone and its contribution to urinary calcium absorption in male rats

**DOI:** 10.1038/srep32085

**Published:** 2016-08-24

**Authors:** Po-Han Lin, Cai-Yun Jian, Jou-Chun Chou, Chien-Wei Chen, Chih-Chieh Chen, Christina Soong, Sindy Hu, Fu-Kong Lieu, Paulus S. Wang, Shyi-Wu Wang

**Affiliations:** 1 Institute and Department of Physiology, School of Medicine, National Yang-Ming University, Taipei 11221, Taiwan, Republic of China; 2 Medical Center of Aging Research, China Medical University Hospital, Taichung 40402, Taiwan, Republic of China; 3 Department of Rehabilitation, Cheng Hsin General Hospital, Taipei 11212, Taiwan, Republic of China; 4 Aesthetic Medical Center, Department of Dermatology, Chang Gung Memorial Hospital, Taoyuan 33378, Taiwan, Republic of China; 5 Department of Medicine, College of Medicine, Chang Gung University, Taoyuan 33302, Taiwan, Republic of China; 6 Department of Biotechnology, College of Health Science, Asia University, Taichung 41354, Taiwan, Republic of China; 7 Department of Medical Research and Education, Taipei Veterans General Hospital, Taipei 11217, Taiwan, Republic of China; 8 Department of Physiology and Pharmacology, College of Medicine, Chang Gung University, Taoyuan 33302, Taiwan, Republic of China

## Abstract

The aim of this study was to investigate the involvement of androgen, mainly testosterone, in the expression of renal senescence marker protein-30 (SMP30) in male rats. We found that the renal SMP30 expression was up-regulated by endogenous testosterone stimulation during puberty. Interestingly, androgen-deficient orchidectomized (ORX) rats exhibited lower SMP30 mRNA and protein expression in the kidney, and that was restored by testosterone propionate (TP) replacement. Abrogation of androgen receptor (AR) activity by co-treatment with flutamide abolished testosterone-induced SMP30 expression in the kidney as well as in the NRK52E cells. However, SMP30 expression was unaltered in the liver of ORX rats. We also showed a positive correlation between renal SMP30 expression and plasma testosterone level during the aging process. TP-induced SMP30 expression in ovariectomized (OVX) rats was observed and was an evidence to explain the gender difference of SMP30 levels. Immunofluorescence assay showed that renal SMP30 was specifically expressed in the proximal tubular segments of the kidney. The urinary Ca^2+^ level was increased in both ORX and male aging rats. Taken together, our results indicate a novel role of testosterone in regulating SMP30 expression specifically in the kidney to contribute to urinary calcium absorption.

Aging is characterized by environmental or physiological pressures to trigger cellular senescence and disturb body homeostatic mechanism, leading to a progressive functional loss of physiological integrity^1^. The peak level of plasma testosterone appears in the second to third decade of life and subsequently declines progressively with aging^2^. Longitudinal studies have suggested that rapid bone loss after 65 year of age in men, which is related to deficient levels of testosterone or estradiol^3^. Elderly men with osteoporosis have higher incidence of fracture and mortality rate than women^4^. An epidemiologic study has indicated that elderly men with high urinary calcium excretion are associated with higher frequency of reduction in bone mineral density (BMD)^5^. Therefore, maintaining calcium homeostasis in the kidney is a way to protect calcium loss from bone osteoclastogenesis. A study has reported that aging-related alteration in the kidney could lead to imbalance of calcium level, which in turn causes multiple Ca^2+^ -regulatory disorders^6^. However, the effect of testosterone on regulating calcium homeostasis in the kidney is still unclear.

Senescence marker protein-30 (SMP30), also known as regucalcin, is initially identified in 1978 as a novel calcium-binding protein without the typical EF-hand motif of calcium binding domain^7^,^8^. Subsequently, analysis of regucalcin has shown that expression of this protein declined in the liver of aged rat; regucalcin was subsequently named as SMP30^9^. The *smp30* gene is highly conserved in vertebrate species, including human, mouse, and rat, and is expressed in various tissues, most prominent in the liver and kidney^10^,^11^,^12^. Previous studies reported specific deposition of lipofuscin and senescence associated β-galactosidase (SA-β-GAL) in renal tubular epithelia, and also decrease in body-weight and lifespan in the SMP30-kncokout mice that all appears distinct aging-associated characteristics^13^,^14^. Hence, SMP30 deficiency has been suggested as a marker of aging. Interestingly, it has been shown that SMP30 expression decreased with aging is an androgen-independent regulation in the liver^15^. However, the role of testosterone in regulating SMP30 expression in the kidney remains unclear.

In the present study, we investigated the effect of testosterone on SMP30 expression in rat kidney. Our results demonstrated that testosterone has a role in stimulating SMP30 mRNA and protein expression through androgen receptor (AR) in rat kidney, which contributes to regulate urinary Ca^2+^ concentration. These findings present a novel role of testosterone in the regulation of SMP30 expression.

## Results

### Involvement of puberty-evoked testosterone in regulating renal SMP30 expression

**Figure 1:**
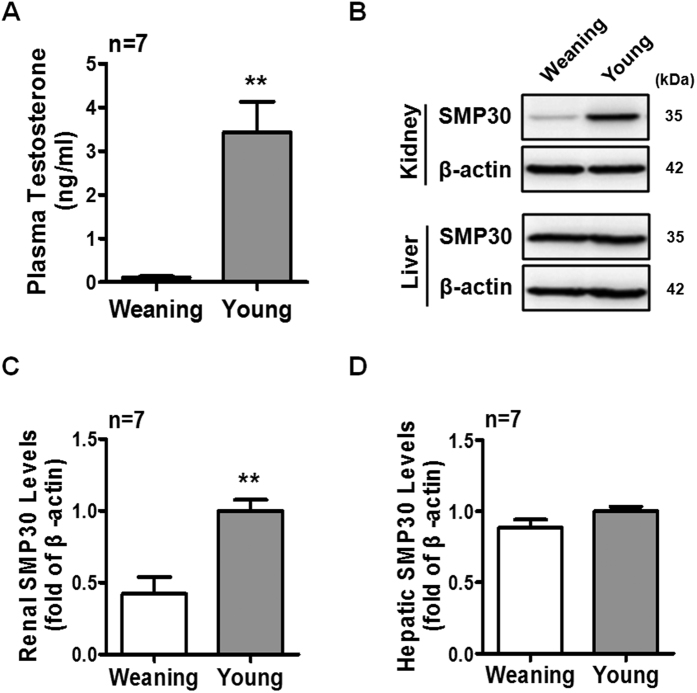
Effects of testosterone on SMP30 expression in rat kidney and liver during puberty. (**A**) Plasma testosterone levels were analyzed by RIA. (**B**) SMP30 protein levels in rat kidney and liver were detected by Western blot analysis, normalized to β-actin expression in the kidney (**C**) and liver (**D**). Data represent means ± SEM (n = 7). **P < 0.01 as compared with the weaning rats. The gels have been run under the same experimental conditions and cropped blots were shown. The entire of membrane pictures of Fig. 1B were presented in the Supplementary Fig. S3.

The process of male sexual maturation, which promotes children’s body maturation into adult, is associated with testicular enlargement and increasing circulating testosterone level. Hence, we first examined whether endogenous testosterone played a role in regulating SMP30 expression in rat kidney and liver during puberty. We found that the puberty-associated enhancement of plasma testosterone was observed in young (2–3 months old) rats (Fig. 1A). Interestingly, the renal SMP30 protein level was increased in young rats (Fig. 1B,C), but was not affected in the liver (Fig. 1B,D), which suggested that endogenous testosterone contributes to SMP30 expression in the kidney during puberty.

### Effects of ORX and TP replacement on SMP30 expression in rat kidney and liver

**Figure 2:**
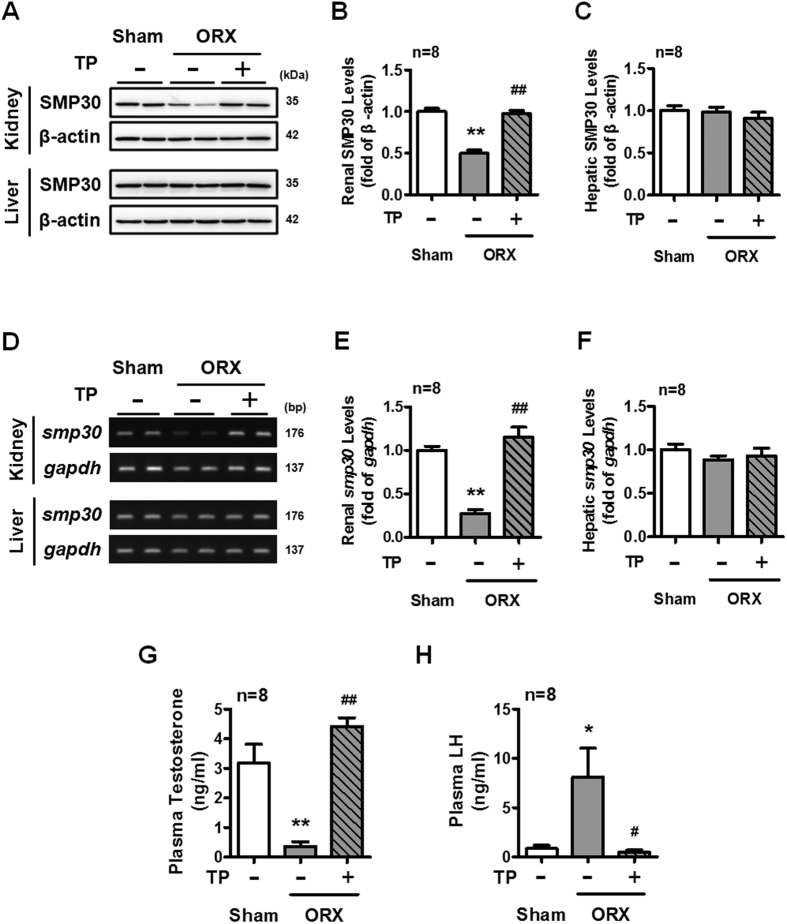
Effects of ORX and TP replacement on SMP30 expression in rat kidney and liver. Two-month-old male rats underwent sham-operation (Sham) or ORX for 7 days and the ORX rats were then treated with sesame oil or TP for another 7 days. (**A**) SMP30 protein expression in rat kidney and liver were analyzed by Western blotting, normalized to β-actin expression in the kidney (**B**) and liver (**C**). (**D**) SMP30 mRNA expression in rat kidney and liver were analyzed by RT-PCR, normalized to *gapgh* expression in rat kidney (**E**) and liver (**F**). Rat plasma testosterone (**G**) and LH (**H**) were measured by RIA. Data represent means ± SEM (n = 8). *P < 0.05, **P < 0.01 as compared with the sham-operated rats; ^#^*P* < 0.05, ^##^*P* < 0.01 as compared with the ORX rats. The gels have been run under the same experimental conditions and cropped blots/gels were shown. The entire of membrane/gels pictures of Fig. 2A,D were presented in the Supplementary Figs S4 and S5.

To further verify whether testosterone plays an important role in regulating SMP30 expression in rat kidney, the effects of ORX and TP replacement on SMP30 expression in rat kidney and liver was examined. Interestingly, we found that ORX rats showed decreased protein expression of SMP30 in the kidney, which was restored by TP replacement (Fig. 2A,B). However, ORX rats did not show any alteration of SMP30 protein expression levels in the liver (Fig. 2A,C). In mRNA levels, SMP30 expressions were observed to be parallel with protein levels in both kidney and liver of ORX rats (Fig. 2D–F). To confirm that testosterone was completely eliminated in ORX rats, the concentrations of plasma testosterone and LH were measured. The plasma testosterone level was significantly reduced in ORX rats, and this reduction was restored by TP replacement (Fig. 2G). Following the negative feedback regulation, the opposite results were shown in the LH levels (Fig. 2H). Taken together, these results suggested that testosterone could regulate renal SMP30 expression at both the mRNA and protein levels.

### Role of AR activation in the testosterone-induced increases of SMP30 protein expression in kidney

**Figure 3:**
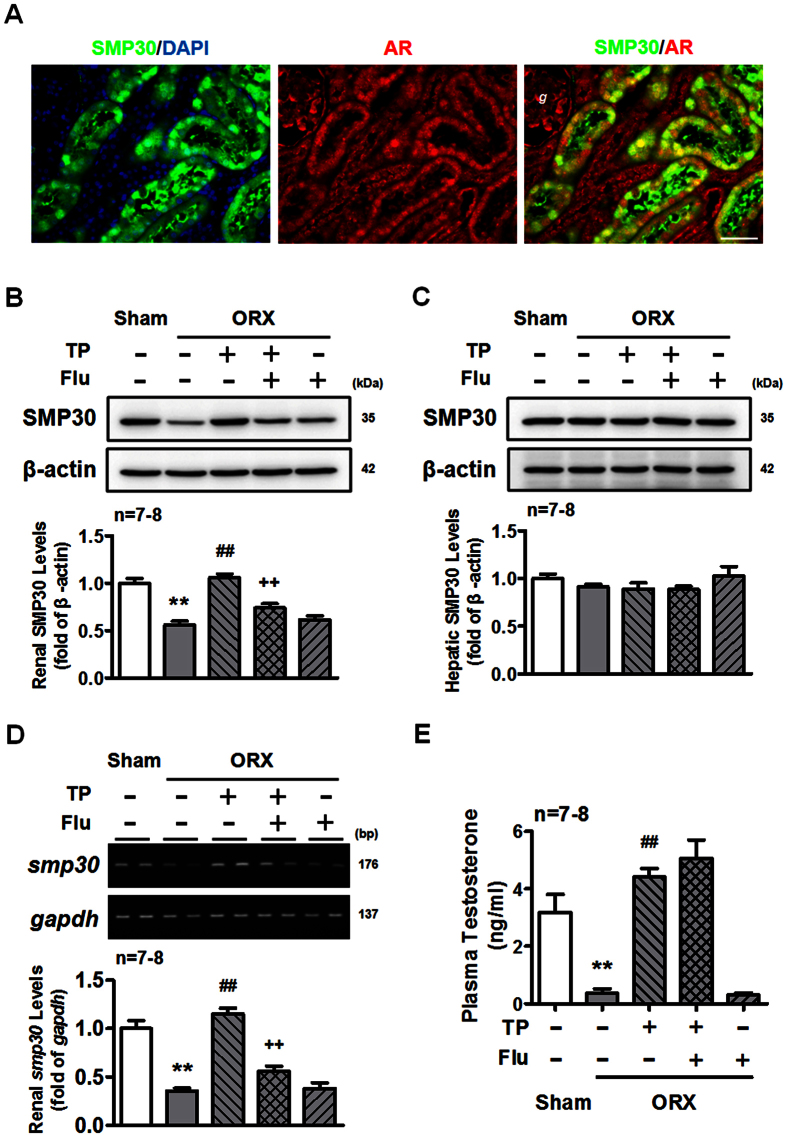
Renal SMP30 expression is androgen-dependent. (**A**) Fluorescence microscopy of double labeled two-month-old male rat kidney sections using goat anti-SMP30 (green) and rabbit anti-AR (red) antibodies. DAPI (blue) was used to counterstain the nuclei. Overlay was presented. (magnification 400x; scale bar = 50 μm). *g*, glomerulus. Two weeks of orchidectomized (ORX) and TP, TP plus flutamide (Flu), and Flu replaced rats were scarified. Kidney (**B**) and liver (**C**) tissues were harvested for Western blot of SMP30 normalized to β-actin expression. (**D**) RT-PCR of SMP30 mRNA expression was performed in the kidney, normalized to *gapdh* expression. (**E**) Plasma testosterone was measured by RIA. Data represent means ± SEM (n = 7–8 each). **P < 0.01 as compared with the sham-operated rats; ^##^*P* < 0.01 as compared with the ORX rats; ^++^*P* < 0.01 as compared with the ORX + TP rats. The gels have been run under the same experimental conditions and cropped blots/gels were shown. The entire of membrane/gels pictures of Fig. 3B–D were presented in the Supplementary Figs S6 and S7.

We further investigated the involvement of AR in testosterone-induced increased SMP30 protein expression in the kidney. The results showed that the expression of SMP30 in renal tubular segment was co-existed with AR (Fig. 3A). Therefore, the role of AR in regulating renal SMP30 expression was evaluated by co-treating ORX rats with TP and an AR antagonist, flutamide, to compete for the ligand binding site of AR. We found that TP-induced increased renal SMP30 protein expression in ORX rats was abolished by co-treatment with flutamide (Fig. 3B). In contrast, there was no alteration in SMP30 expression in the liver of ORX rats (Fig. 3C). The result of renal SMP30 mRNA levels was paralleled with the protein levels in ORX rats co-treated with TP and flutamide (Fig. 3D). The plasma testosterone was measured to confirm that reduction of testosterone was restored by TP replacement in the ORX rats (Fig. 3E). The effect of testosterone on renal SMP30 expression was also evaluated in normal rat kidney epithelial NRK52E cells. Treatment of the cells with testosterone increased SMP30 expression (Supplementary Fig. S1A,B). In addition, testosterone-induced SMP30 expression was also blocked by flutamide in the NRK52E cells (Supplemental Fig. S1C,D). These results suggested that testosterone regulated renal SMP30 expression via AR mediation at transcription and translation levels.

### Aging-associated reduction of plasma testosterone is correlated with reduction of SMP30 expression in rat kidney

**Figure 4:**
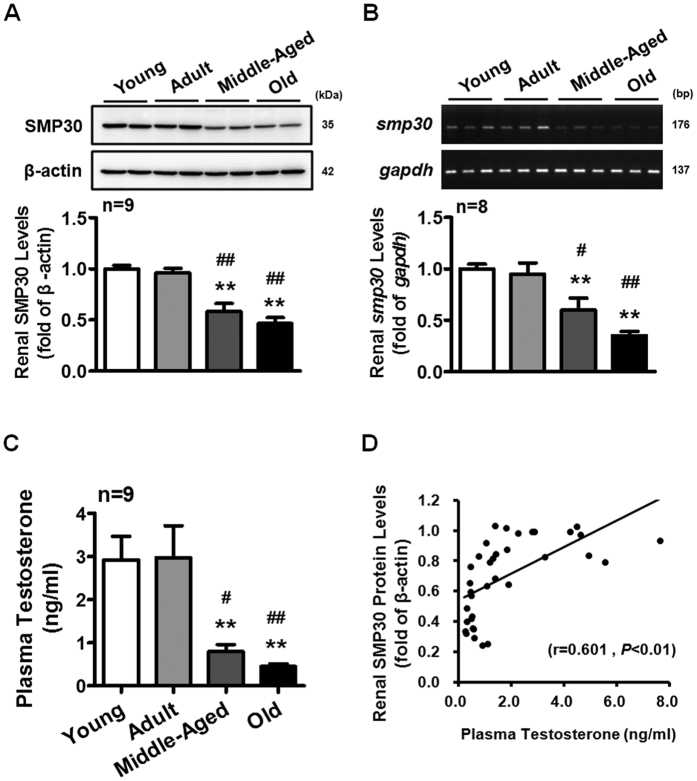
Aging effects on SMP30 expression in rat kidney. Male rats were divided into four groups: young (2–3 months), adult (5–6 months), middle-aged (12–15 months), and old (20–24 month). (**A**) SMP30 protein expression in rat kidney (n = 9) was analyzed by Western blot, normalized to β-actin expression in the kidney. (**B**) SMP30 mRNA expression in rat kidney (n = 8) was analyzed by RT-PCR, normalized to *gapdh* expression in the kidney. (**C**) Plasma testosterone levels were measured by RIA (n = 9). (**D**) Pearson correlation coefficients were used to evaluate the relationship between plasma testosterone and renal SMP30 protein expression (n = 36; Pearson: r = 0.601, *P* < 0.01). Data represent means ± SEM. **P < 0.01 as compared with the young rats; ^#^*P* < 0.05, ^##^*P* < 0.01 as compared with the adult rats; ^+^*P* < 0.05 as compared with the middle-aged rats. The gels have been run under the same experimental conditions and cropped blots/gels were shown. The entire of membrane/gels pictures of Fig. 4A,B were presented in the Supplementary Fig. S8.

Based on the above results, the relationship between SMP30 protein expression and plasma testosterone during aging was further analyzed. We found that the renal SMP30 protein and mRNA levels were decreased in the middle-aged (12–15 months old) and old (20–24 months old) rats (Fig. 4A,B). The plasma testosterone level was also reduced with age (Fig. 4C). Therefore, the renal SMP30 expression levels positively correlated with plasma testosterone levels in the kidney (Pearson: r = 0.601, *P* < 0.01) (Fig. 4D).

### Testosterone level causes gender-related difference in renal SMP30 protein expression

**Figure 5:**
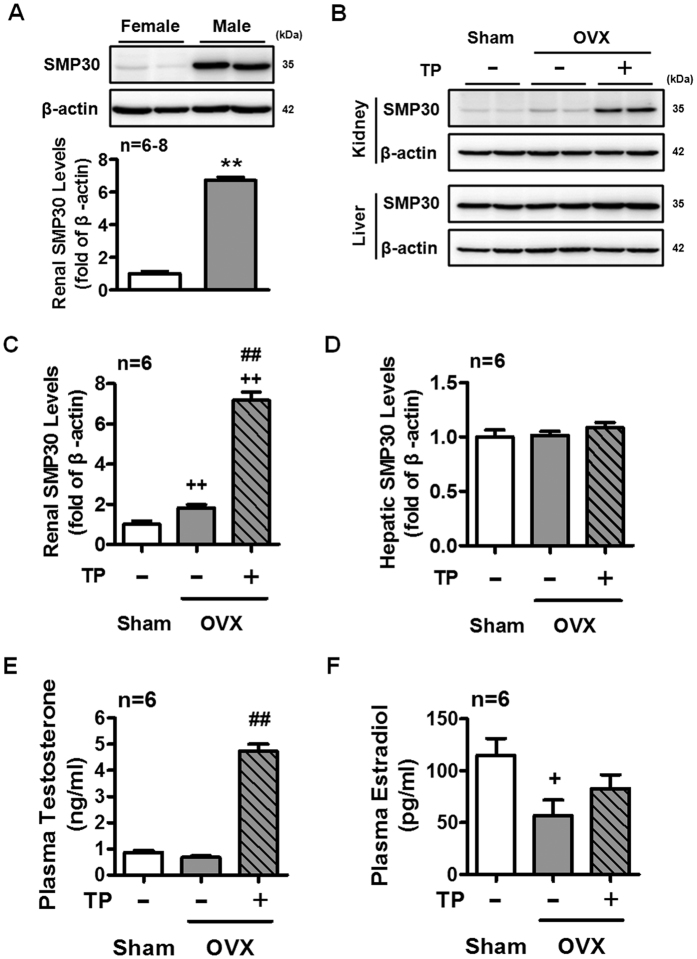
Effects of TP treatment on SMP30 protein expression in the kidney and liver of OVX rat. (**A**) Gender difference in protein levels of SMP30 in rat kidney. The renal SMP30 protein levels in two-month-old of both female (n = 8) and male (n = 6) rats were analyzed by Western blot, normalized to β-actin expression. Two-month-old female rats underwent sham-operation (Sham) or OVX for 7 days and the OVX rats were then treated with sesame oil or TP for another 7 days. (**B**) Kidney (n = 6) and liver (n = 6) tissues were harvested for Western blot of SMP30, normalized to β-actin expression in the kidney (**C**) and liver (**D**). Plasma testosterone (**E**) and estradiol (**F**) levels were measured by RIA (n = 6). Data represent means ± SEM. **P < 0.01 as compared with female rats; ^+^*P* < 0.05, ^++^*P* < 0.01 as compared with sham rats; ^##^*P* < 0.01 as compared with the OVX rats. The gels have been run under the same experimental conditions and cropped blots were shown. The entire of membrane pictures of Fig. 5A,B were presented in the Supplementary Fig. S9.

A previous study has reported that the *smp30* gene is located in the X chromosome of rodent and human^9^. We further investigated whether the expression level of SMP30 in the kidney was associated with gender difference. Interestingly, the renal SMP30 protein expression level in the male rats was higher than in the female rats at the same age (2-month old) (Fig. 5A). Next, female rats were withdrawn sexual hormones by ovariectomy (OVX) and were further treated with TP to investigate the role of testosterone in regulating SMP30 protein expression in the kidney of OVX rats. The results showed that TP treatment significantly increased SMP30 protein expression and that was also slightly increased in the kidney of OVX rats, but no alteration of SMP30 expression levels in the liver (Fig. 5B–D). Treatment of TP in the OVX rats showed a significant high plasma testosterone level as compared with untreated OVX rats (Fig. 5E). However, the concentration of plasma estradiol was not altered by TP treatment in the OVX rats (Fig. 5F). Taken together, these results suggested that testosterone specifically up-regulated SMP30 expression in the kidney, which might contribute to gender difference in renal SMP30 expression.

### Localization of SMP30 in rat kidney

**Figure 6:**
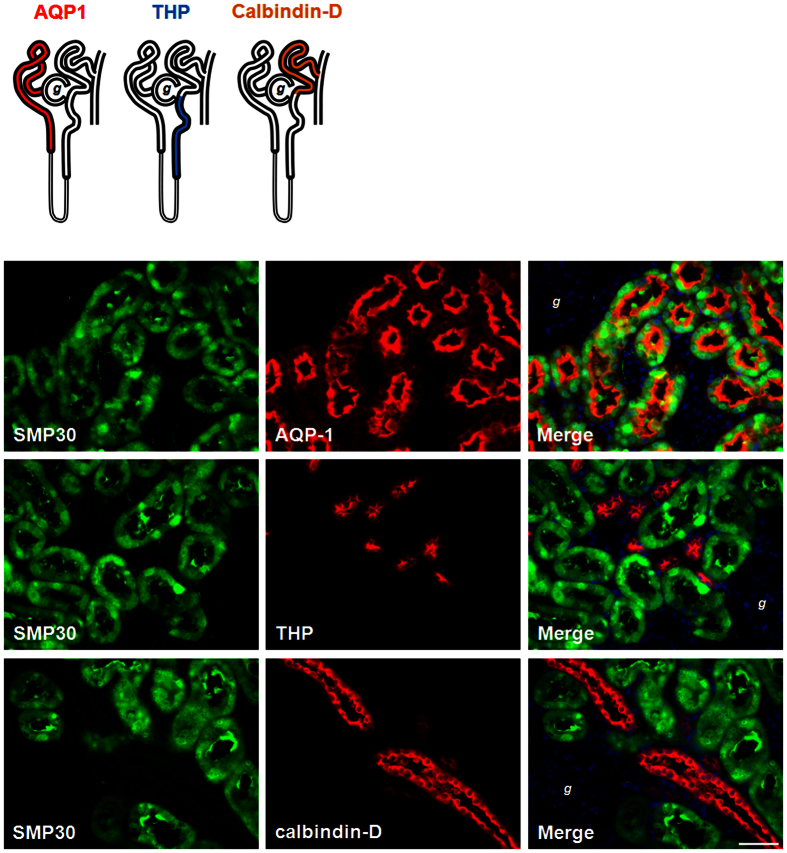
Immunolocalozation of SMP30 expression in rat kidney. Kidney tissues were harvested from two-month-old male rats. Tissues were formalin-fixed and paraffin-embedded. Four micrometer cross sections of the tissues were collected onto slides. Fluorescence immunohistochemical staining of kidney sections with goat anti-SMP30, mouse anti-aquaporin-1 (AQP1, renal proximal tubule marker protein), rabbit anti-Tamm-Horsfall glycoprotein (THP, the thick ascending limb of the loop of Henle marker protein), and rabbit anti-calbindin-D (renal distal tubule marker protein) antibodies were used to evaluate SMP30 localization. Nuclei counterstaining with 4,6-diamidino-2-phenylindole (DAPI) was used. Overlay was presented. (magnification 400x; scale bar = 50 μm). *g*, glomerulus.

It was noted that SMP30 was not fully expressed in all renal tubular segments (Fig. 2A). Therefore, we further identified SMP30 expression in specific tubular segments of the rat kidney. Double immunofluorescence staining of renal sections was performed using an anti-SMP30 antibody combined with anti-aquaporin-1 (AQP1, as the renal proximal tubule marker protein), anti-Tamm-Horsfall glycoprotein (THP, as the thick ascending limb of the loop of Henle marker protein), or anti-calbindin-D (as the renal distal tubule marker protein) antibodies, respectively. We found that SMP30 protein was shown to colocalizate with the AQP1 protein, but not with THP or calbindin-D (Fig. 6), suggesting that renal SMP30 is specifically expressed in renal proximal tubular segments.

### Testosterone-enhanced renal SMP30 expression is associated with PMCA4 expression and contribution to urinary calcium level

**Figure 7:**
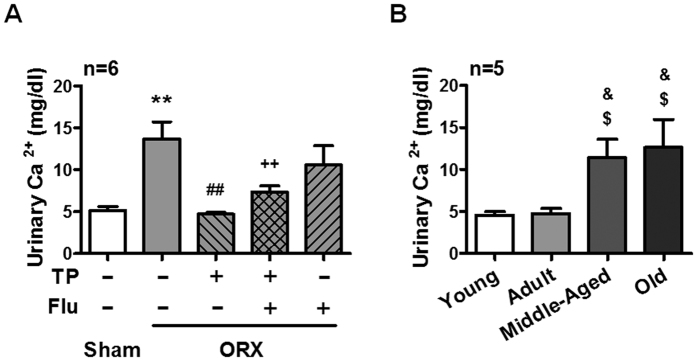
Effects of testosterone on urinary Ca^2+^ level. (**A**) Two weeks of orchidectomized (ORX) and TP, TP plus flutamide (Flu), and Flu replaced rats, and (**B**) aged male rats were housed in metabolic cages for 24 h and urine was collected for Ca^2+^ analysis. Data represent means ± SEM. **P < 0.01 as compared with the sham-operated rats; ^##^*P* < 0.01 as compared with the ORX rats; ^++^*P* < 0.01 as compared with the ORX + TP rats; ^$^*P* < 0.05 as compared with young rats; ^&^*P* < 0.05 as compared with adult rats.

A previous study has demonstrated that SMP30/regucalcin stimulates Ca^2+^ -ATPase activity and increases Ca^2+^ uptake in the basolateral membranes of rat kidney cortex^16^. Among the four plasma membrane Ca^2+^ ATPase (PMCA) isoforms, the PMCA1 and PMCA4 isoforms are expressed ubiquitously including in kidney^17^,^18^. We further investigated whether testosterone-induced SMP30 expression has a role in regulating plasma membrane Ca^2+^ ATPase expression. The results showed that treatment with testosterone increased PMCA4 expression and this stimulatory effect was abolished by flutamide in the NRK52E cells. However, treatment with testosterone did not affect PMCA1 expression (Supplementary Fig. S2A,B). Moreover, it has also been shown that SMP30 enhances Ca^2+^ efflux from renal epithelial cells^19^, suggesting that renal SMP30 might be involved in regulating urinary Ca^2+^ homeostasis. To clarify this issue, we further investigated whether testosterone-induced renal SMP30 expression had a role in regulating urinary Ca^2+^ homeostasis. Importantly, the urinary Ca^2+^ level was significantly increased in the ORX rats, and this effect was reduced by treating the ORX rats with TP. Moreover, testosterone replacement reduction of urinary Ca^2+^ level was abolished by co-treating the ORX rats with TP and flutamide (Fig. 7A). However, there was no difference in the plasma Ca^2+^ level, volume of urine, water intake and plasma parathyroid hormone (PTH) among all the five groups of rats tested (Supplementary Table S1). To further confirm that this effect is a naturally occurring event during aging, the urinary calcium level was measured in the male aging rat model. We found that the urinary Ca^2+^ level was increased in the middle-aged (12–15 months old) and old (20–24 months old) rats (Fig. 7B). Interestingly, there was also no difference in the plasma Ca^2+^ level, volume of urine, water intake and plasma PTH among all the four groups (Supplementary Table S2). These results suggested that testosterone enhanced renal SMP30 expression participates in the regulation of urinary calcium and might be involved in the maintenance of Ca^2+^ homeostasis.

## Discussion

It has been reported that SMP30 is significantly expressed in the liver and kidney in humans and rodents^11^,^12^. Fujita and colleagues previously demonstrated that the reduction of SMP30 expression with age in rat liver is an androgen-independent regulation^15^. However, possible involvement of testosterone in regulating SMP30 expression in the kidney is still unclear. In the present study, we found that endogenous testosterone-evoked since puberty up-regulated SMP30 expression in the rat kidney. Moreover, compelling evidences were presented that the expression of SMP30 was significantly decreased in the kidney of androgen-deficient ORX rats, and this reduction was restored by replacement of testosterone propionate (TP) through AR. The results presented a positive correlation between the renal SMP30 expression and plasma testosterone level during aging. Testosterone significantly induced SMP30 expression in the kidney of the OVX rats, which might explain the gender difference in SMP30 expression in rat kidney. Importantly, findings of this work suggest that testosterone-induced SMP30 expression in the kidney is associated with the regulation of urinary Ca^2+^ level. To our knowledge, this is a first demonstration that testosterone regulates SMP30 expression in rat kidney through AR to maintain urinary Ca^2+^ level.

Although it was shown that dihydrotestosterone (DHT) down-regulated SMP30/regucalcin mRNA expression in prostate cancer LNCaP cells, this inhibitory effect was abolished by flutamide, an AR antagonist, suggesting that down-regulation of SMP30/regucalcin expression by DHT is through AR modulation^20^. In the present study, results suggested that testosterone, an androgen, was involved in up-regulating SMP30 expression in rat kidney. This observation was supported by the evidence that testosterone-evoked since puberty increased SMP30 expression in rat kidney, whereas SMP30 protein in the liver was not altered. Moreover, TP treatment significantly increased SMP30 expression in the kidney rather than the liver of the ORX rats. Our results also showed that TP-induced upregulated SMP30 expression in rat kidney was abrogated by co-treatment with flutamide,suggesting that activation of AR might be involved in testosterone-induced SMP30 expression in rat kidney. Expression of SMP30 in the kidney was previously shown to gradually decrease during aging^21^. In the present study, our results indicated a positive correlation between plasma testosterone and SMP30 protein level in rat kidney.

To explain the gender difference on renal SMP30 expression, the effect of testosterone on the SMP30 expression in OVX rats was further examined. The expression of SMP30 protein in the kidney of the OVX rats was significantly increased by TP treatment, whereas SMP30 protein in the liver of the OVX rat was not altered. The results of plasma testosterone and estradiol assays suggested that enhanced SMP30 expression in rat kidney is specifically through the action of testosterone rather than that of estradiol. Moreover, we also found that SMP30 protein level increased in the OVX female rat group as compared with the sham-operated rats. In agreement with our findings, Kurota and Yamaguchi reported that estrogen down-regulated SMP30 expression in rat kidney^22^; Ueoka and Yamaguchi also reported that estrogen did not affect SMP30 expression in the liver of OVX rats^23^. Therefore, these results suggested that testosterone and estradiol have opposing regulatory properties in renal SMP30 expression.

The effect of calciotropic hormones on the expression of SMP30/regucalcin in the kidney has been investigated. It has been shown that administration of calcitonin or parathyroid hormone (PTH) in thyroparathyroidectomized male rats with calcium did not cause any alteration on the SMP30 mRNA expression in the kidney^24^. Interestingly, SMP30 mRNA expression in normal rat kidney epithelial NRK52E cells was increased by PTH, but not by calcitonin or 1,25-dihydroxyvitamin D3^25^. However, the results from the present study showed that plasma PTH levels did not cause any alteration in SMP30 expression in the ORX rats as well as in the male aged rats, suggesting that down-regulation of SMP30 in the kidney of ORX or aging rats was not regulated by PTH.

Kidney plays a physiological role in the maintenance of Ca^2+^ homeostasis. Approximately 50% of Ca^2+^ in the plasma is ionized and filtered by glomerulus. Normally, 99% of filtered Ca^2+^ is reabsorbed and less than 5% appears in the urine. In the nephron, the proximal tubule reabsorbs over half of the filtered Ca^2+^ via the primary paracellular pathway. Another 15% is reabsorbed in the ascending limb of the loop of Henle; about 10 to 15% is delivered by the distal tubule. In distal tubule, the Ca^2+^ reabsorption occurred trough a transcellular route that across the luminal membrane by a permeable ion channel (e.g., TRPV5) and then delivered by intracellular Ca^2+^ -binding protein (e.g., calbindin-D_28k_) to the basolateral membrane^26^. It has been reported that testosterone is involved in regulating of urinary Ca^2+^ level. Couchourel and colleagues demonstrated that testosterone enhanced Ca^2+^ reabsorption by activating the T-type Ca^2+^ channel in the distal tubular luminal membrane^27^. In the present study, results showed that ORX operation reduced SMP30 expression in rat kidney and caused an increase of urinary Ca^2+^ level. Moreover, the urinary Ca^2+^ level was also increased in male rats during aging. In contrast to our results, the opposite effect of testosterone was demonstrated by Hsu and colleagues who reported that testosterone enhanced urinary Ca^2+^ excretion by inhibiting expression of renal Ca^2+^ receptor TRPV5 and transport protein calbindin-D_28K_ in mice^28^, indicating involvement of testosterone in urinary Ca^2+^ excretion by regulating distal tubular associated protein expression. Although it is still unclear whether any molecular or chemical events might be involved in urinary Ca^2+^ regulation after ORX in both mice and rats, the present study suggested that testosterone might affect urinary Ca^2+^ reabsorption through regulating renal SMP30 expression.

Intracellular Ca^2+^ overload has been reported to cause cytotoxicity and result in apoptotic cell death^29^,^30^. In the present study, the immunofluorescence analysis showed that SMP30 protein was specifically expressed at the renal proximal tubular segments. As mentioned above, the proximal tubule is the main section of the tubule where Ca^2+^ reabsorption took place via paracellular pathway. However, some reports have indicated that the Ca^2+^ pump exists in proximal tubular cells, where absorption of a smaller fraction of Ca^2+^ through intracellular pathway does occur^17^,^31^. This raises the question on how SMP30 is involved in regulating Ca^2+^ reabsorption in the proximal tubule. It has been shown that SMP30 enhances Ca^2+^ efflux from renal epithelial cells^19^. In the present study, results showed that testosterone-increased SMP30 expression was associated with PMCA4 expression in the NRK52E cells. Inoue and colleagues also reported that overexpression of SMP30 prevented cell death by Ca^2+^ superfluous influx-mediated in the proximal tubular epithelial LLC-PK1 cells^19^. Moreover, overexpression of SMP30/regucalcin has been reported suppresses apoptotic cell death induced by Bay K 8644, an agonist of L-type Ca^2+^ channel, via up-regulation of Bcl-2 mRNA expression^32^, suggesting that SMP30 might play an important role in self-defense against Ca^2+^ superfluous influx-induced apoptosis. Moreover, overexpression of SMP30 also enhances expression of the tight junction protein ZO-1 in HepG2 cells, implying that SMP30 contributes the cell-to-cell interactions^33^. The above evidences implies that down-regulation of SMP30 causes an imbalance of intracellular Ca^2+^ levels, resulting in apoptosis and damage of integrity of tight junction, which in turn reduces Ca^2+^ absorption in the proximal tubule.

In conclusion, this study showed that SMP30 expression was androgen-dependent in rat kidney. Testosterone deficiency reduced SMP30 expression in the kidney, which correlated to increases in urinary Ca^2+^. Based on the results of this study, we propose a new insight that reduction of renal SMP30 protein expression in the aging process with high urinary calcium excretion might be correlated to the osteoporosis.

## Methods

### Animal experiments

Sprague-Dawley rats were purchased from the Laboratory Animal Center of National Yang-Ming University (Taipei, Taiwan). Rats were housed in a 14 h artificial illumination (0600–2000) and temperature (22 ± 2 °C) controlled room. Food and water were given *ad libitum*. All animal experimental protocols were according to the guide for the care and use of laboratory animals (8^th^ edition) and all experimental protocol was approved by the Institutional Animal Care and Used Committee (IACUC), National Yang-Ming University, Taipei, Taiwan (Permit Number: 1021226).

#### Experiment I-Aging model

Male rats were divided into 4 groups with the age of 2–3 (young), 5–6 (adult), 12–15 (middle-aged), and 20–24 (old) months. Moreover, the age of 3 weeks male rats were defined as the weaning phase in the present study.

#### Experiment II-Orchidectomized (ORX) model

Two to three months old male rats underwent bilateral ORX or sham operation under anesthesia with sodium pentobarbital (40 mg/kg; Koch-Light. Lab. Ltd., Colnbrook, Bucks, England). After 1-week recovery, the rats were divided into three and/or five groups: **1.** sham-operated (Sham) rats were subcutaneously injected with sesame oil (Sigma-Aldrich, St Louis, MO, USA) as a control; **2.** ORX rats were subcutaneously injected with sesame oil; **3.** ORX rats were treated with testosterone propionate (TP, 2 mg/kg; Fluka, Buchs, Switzerland) (ORX + TP) via subcutaneous injection; **4.** ORX rats were subcutaneously injected with TP combined with flutamide (Flu, 6 mg/kg; Sigma-Aldrich, St Louis, MO, USA) (ORX + TP + Flu); **5.** ORX rats were subcutaneously injected with Flu (ORX + Flu) once daily for 7 days.

#### Experiment III-Ovariectomized (OVX) model

Two to three months old female rats underwent sham or bilateral OVX operation under anesthesia with sodium pentobarbital (40 mg/kg). After 1-week recovery, rats were divided into three groups: **1.** sham-operated (Sham) rats were subcutaneously injected with sesame oil as a control; **2.** OVX rats were subcutaneously injected with sesame oil; **3.** OVX rats were treated with testosterone propionate (TP, 2 mg/kg) (OVX + TP) via subcutaneous injection once daily for 7 days.

All experimental rats were sacrificed using sodium pentobarbital anesthesia and exsanguinations via the abdominal aortic artery. Blood samples were collected. Subsequently, kidneys and livers were quickly excised, immediately immersed in liquid nitrogen and stored at −80 °C for further analysis.

### Radioimmunoassay (RIA) and enzyme immunoassays of plasma testosterone, estradiol, luteinizing hormone (LH), and parathyroid hormone (PTH)

Plasmas were separated from blood samples after centrifugation at 10,000 *g* for 5 min. The concentration of plasma testosterone, LH, and estradiol were detected by RIA. The sex steroid hormones, including testosterone and estradiol, were measured in RIA as previously described^34^,^35^. The sensitivity of the testosterone RIA with an anti-testosterone serum (No. W8) was 2 pg per assay tube. The intra- and inter-assay coefficients of variation were 4.1% (n = 6) and 4.7% (n = 10), respectively. The sensitivity of the estradiol RIA with an anti-estradiol serum (No. W1) was 1 pg per assay tube. The intra- and inter-assay coefficients of variation were 6.0% (n = 5) and 5.9% (n = 5), respectively. The luteinizing hormone (LH) RIA was performed as previously described^36^. The rat-I-6 used for iodination and the rat LH-RP-3, which served as standard preparations were provided by the National Hormone and Pituitary Program, National Institute of Diabetes and Digestive and Kidney Diseases, National Institute of Child Health and Human Development, and Department of Agriculture, USA. The sensitivity was 0.1 ng for LH RIA. The intra- and interassay coefficients of variability were 3.8% (n = 4), and 6.6% (n = 5), respectively. Plasma PTH concentration was determined by the enzyme immunoassay kit (Phoenix Pharmaceuticals, Burlingame, CA, USA).

### Urinary and plasma Ca^2+^ analysis

In the present study, rats were housed in metabolic cages, one rat in one cage, for 24 h. The urine was collected to determine the concentration of Ca^2+^ using an automatic analyzer (Fuji Dri-Chem 4000i; Fujifilm, Tokyo, Japan). Moreover, the volumes of urine and water intake after 24 h were recorded. Plasma Ca^2+^ concentration was determined by the Fuji Dri-chem 4000i automatic analyzer (Fujifilm, Tokyo, Japan).

### Protein preparation and Western blot analysis

Western blot analyses were employed to determine the protein levels of the kidney and liver as described previously^34^. Briefly, samples were boiled for 5 min with a sample buffer (0.125 M Tris-Cl pH 6.8, 4% SDS, 20% glycerol, 10% 2-mercaptoethanol, 0.1% bromophenol blue) at a ratio of 1:1. Electrophoresis was performed using 12% sodium-dodecyl sulfate (SDS)-polyacrylamide gel. Separated proteins were transferred onto polyvinyl difluoride membranes (Millipore, Billerica, MA, USA). The membranes were blocked with 5% fat-free milk (Anchor, Auckland, NZ) in TBS-T (20 mM Tris, 0.137 M NaCl, 0.1% Tween-20, pH 7.6) at room temperature for at least 1 h. The membranes were incubated with a specific antibody against SMP-30 (1:500; Santa Cruz Biotechnology, Santa Cruz, CA, USA) and β-actin (1:5000; Sigma-Aldrich) overnight at 4 °C. The membranes were then incubated with horseradish peroxidase-conjugated goat anti-mouse and/or donkey anti-goat IgG (1:10000, Jackson ImmunoResearch Laboratories, West Grove, PA, USA) for 1 hr. Subsequently the membranes were developed with enhanced chemiluminescence (PerkinElmer Life Sciences, Boston, MA, USA). To quantify the intensity of the protein expression levels, the signal was recorded by Luminescence Imaging System LAS-4000 (*GE* Healthcare Life Sciences, Pittsburgh, PA, USA), and the band densities were determined as arbitrary absorption units using the Image-J software program.

### RNA isolation and semiquantitative RT-PCR

Total RNAs were isolated from rat kidney and liver tissues with Trizol reagent (Invitrogen-Life Technologies, Grand Island, NY, USA) as described^37^. Two micrograms of total RNA was reverse transcribed to synthesize cDNA using ThermoScript™ RT-PCR System (Life technologies, Grand Island, NY, USA) for RT-PCR. The cDNA samples were diluted ten-fold, two microliters of the diluted sample was used for PCR amplification with SMP30 and GAPDH primers. GAPDH was used as an internal control. The following primers were used: SMP30 5′-GGGAAGCTTTGGGTGGCCTGTT-3′ and 5′-TCGGCGCTCATCCCATCCCT-3′ and GAPDH 5′-CGGCACAGTCAAGGCTGAGAATG-3′ and 5′-TGAAGACGCCAGTAGACTCCACGAC-3′. The PCR parameters comprised a cycle of 5 min at 95 °C, 30 s at 60 °C and 30 s at 72 °C for 30 cycles (in SMP30) and 27 cycles (in GAPDH). The amplified PCR products were separated by electrophoresis on a 1.2% agarose gel and visualized with ethidium bromide.

### Immunofluorescent staining

Kidney tissues were fixed in 10% formalin for 24 h and then dehydrated using serial percentage alcohols (75%, 85%, 90%, and 100%) and embedded in paraffin. Four micrometer cross sections were collected onto slides, then deparaffinized and rehydrated. Antigen retrieval was performed by using a boiling retrieval buffer (10mM sodium citrate, 0.05% Tween-20, pH 6.0). Sections were blocked with 3% BSA (Sigma-Aldrich) for 1 h at room temperature. For SMP30 and androgen receptor (AR) or SMP30 and aquaporin-1 (AQP1) or SMP30 and Tamm-Horsfall glycoprotein (THP) or SMP30 and calbindin-D double immunostaining, goat anti-SMP30 (1:200; Santa Cruz), rabbit anti-AR (1:200; Santa Cruz), anti-THP (1:200; Santa Cruz), or anti-calbindin-D (1:300; Abcam, Cambridge, MA, USA), and mouse anti-AQP1 (1:200; Santa Cruz) antibodies were applied at the same time. After overnight incubation with primary antibodies and rinsed with PBS-T, sections were then incubated with Alexa fluro 488-conjugated donkey anti-goat antibody (1:500; Jackson ImmunoResearch), Alexa fluor 594-conjugated donkey anti-rabbit or anti-mouse antibodies (1:500; Jackson ImmunoResearch) for 2 h. Finally, sections were coverslipped with mounting medium containing DAPI (Vector Laboratories, Burlington, ON, Canada). Images for immunofluorescent analysis were captured with Leica DM 6000B fluorescence microscope (Leica Microsystem, Wetzlar, Germany).

### Statistical analysis

All data were expressed as mean ± standard error of the mean (SEM). Data were analyzed using one-way ANOVA to evaluate the difference among all groups. Student’s unpaired *t*-test was used for comparison between two groups. Statistically significant was assumed at P < 0.05^38^. Statistical analysis was performed using Prism version 5.0 software (GraphPad, San Diego, CA, USA).

## Additional Information

**How to cite this article**: Lin, P.-H. *et al.* Induction of renal senescence marker protein-30 (SMP30) expression by testosterone and its contribution to urinary calcium absorption in male rats. *Sci. Rep.* **6**, 32085; doi: 10.1038/srep32085 (2016).

## Supplementary Material

Supplementary Information
