# Supplementary material for: Induction of renal senescence marker protein-30 (SMP30) expression by testosterone and its contribution to urinary calcium absorption in male rats
Source: Sci Rep. 2016 Aug 24;6:32085. doi: 10.1038/srep32085 (PMC4995462; doi:10.1038/srep32085)
Supplement: Supplementary Information [file srep32085-s1.pdf]

(Revised)

Induction of renal senescence marker protein-30 (SMP30) expression by testosterone and its contribution to urinary calcium absorption in male rats

Po-Han Lin[1], Cai-Yun Jian[1], Jou-Chun Chou[2], Chien-Wei Chen[1], Chih-Chieh Chen[1], Christina Soong[3], Sindy Hu[4,5], Fu-Kong Lieu[3]*, Paulus S. Wang[1,2,6,7]*, Shyi-Wu Wang[4,8]*

[1]Institute and Department of Physiology, School of Medicine, National Yang-Ming University, Taipei 11221;

[2]Medical Center of Aging Research, China Medical University Hospital, Taichung 40402;

[3]Department of Rehabilitation, Cheng Hsin General Hospital, Taipei 11212;

[4]Aesthetic Medical Center, Department of Dermatology, Chang Gung Memorial Hospital, Taoyuan 33378;

[5]Department of Medicine, College of Medicine, Chang Gung University, Taoyuan 33302;

[6]Department of Biotechnology, College of Health Science, Asia University, Taichung 41354;

[7]Department of Medical Research and Education, Taipei Veterans General Hospital, Taipei 11217,

[8]Department of Physiology and Pharmacology, College of Medicine, Chang Gung University, Taoyuan 33302, Taiwan, Republic of China.

*Corresponding authors and persons to whom reprint requests should be addressed:

[1] Paulus S Wang, Ph.D.
Institute and Department of Physiology, School of Medicine, National Yang-Ming University, Taipei 11221, Taiwan, Republic of China.
Tel: +886-2-2826-7082
Fax: +886-2-2826-4049
E-mail: pswang3879@gmail.com

[2] Shyi-Wu Wang, Ph.D.
Department of Physiology and Pharmacology, College of Medicine, Chang-Gung University, Taoyuan 33302, Taiwan, Republic of China.
Tel: +886-3-211-8800 ext.5253
Fax: +886-2-211-8700
E-mail: swwang@mail.cgu.edu.tw

[3] Fu-Kong Lieu, M.D.
Department of Rehabilitation, Cheng Hsin General Hospital, Taipei 11212, Taiwan, Republic of China.
Tel: +886-2-2826-4400 ext.3806
Fax: +886-2-2826-4400 ext.3814
E-mail: ch2276@chgh.org.tw

Supplemental Mertinals and Methods:

NRK52E cell culture

Normal rat kidney epithelial NRK52E line was purchased from the Bioresource Collection and Research Center (BCRC, Hsinchu, Taiwan). NRK52E cells were maintained in DMEM medium (Gibco Laboratories, Grand Island, NY, USA) supplemented with 5% fetal bovine serum (FBS, Hyclone, *GE* Healthcare Life Sciences), penicillin G (100 IU/ml; Sigma-Aldrich), streptomycin (100 μg/ml; Sigma-Aldrich), sodium bicarbonate (1.5 g/L; Sigma-Aldrich), glucose (4.5g/L; affymetrix USB, Santa Clara, CA, USA) and L-Glutamine (4 mM; Sigma-Aldrich) in a humidified incubator (37℃, 5% CO_2).

For the exprериments, NRK52E cells had grown to confluence, culture medium was removed and rinsed twice with 1x PBS, following cells were rested by incubation in serum- and phenol red-free DMEM (Gibco) medium for 12 h before the beginning of treatment. The condtional medium was then replaced and incubation for the times indication. The detailed procedures were described in the legends.

Western blot analysis for NRK52E cell

The process of protein lysate was silimar to the tissue samples and SDS-page preparation was described in the methods of manuscript. Primary antibodies used were mouse anti-SMP30 (1:350), rabbit anti-PMCA1 (1:300) and goat anti-PMCA4 (1:300) antibodies (Santa Cruz), and sencondary antibodies were biotinylated goat anti-mouse IgG (1:2000), goat anti-rabbit IgG (1:2000) and rabbit anti-goat IgG (1:2000) (Vector Laboratories, Burlinggame, CA, USA). Binding sensitivity was enhanced by using ABC solution (Standard VECTASTAIN ABC Kit; Vector Laboratories). Subsequently the membranes were developed with enhanced chemiluminescence (PerkinElmer Life Sciences). To quantify the intensity of the protein expression levels, the signal was recorded by Luminescence Imaging System LAS-4000 (*GE* Healthcare Life Sciences).

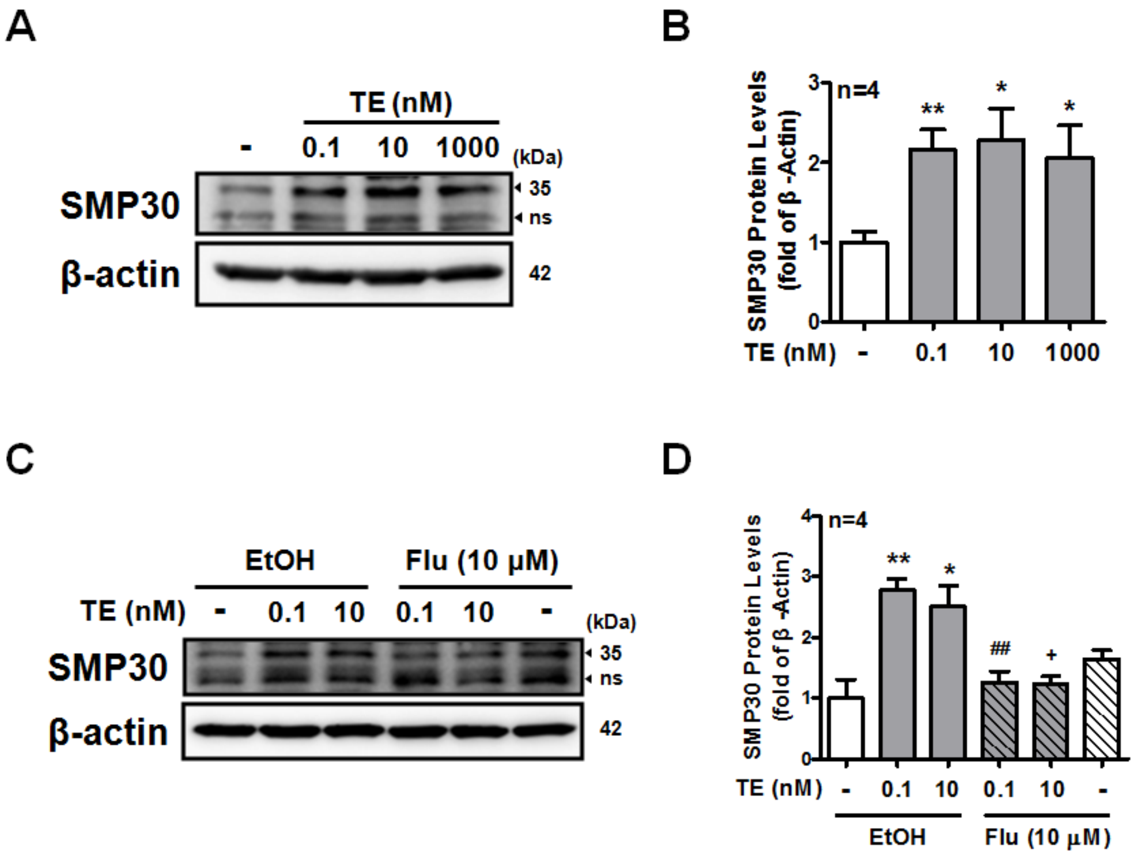

Supplementary Fig. S1. Involvement of the AR in the testosterone-induced SMP30 expression in NKR52E cells. (A) After resting for 12 h, NRK52E cells were treated with testosterone (0.1, 10, 1000 nM) (Sigma-Aldrich), or absolute alcohol (EtOH) as control vehicle, in serum- and phenol red-free DMEM medium for next 12 hr. At the end of time, cells were collected and the expression of SMP30 protein level was analyed by Western blot, and (B) the quantitative analysis of SMP30 in normalization to β-actin was shown. (C) Cells were pretreated with flutaminde (10 μM) for 1 h and then treated with testosterone (0.1 and 10 nM) or EtOH in serum- and phenol red-free DMEM medium for 12 h. At the end of time, cells were collected and the expression of SMP30 protein level was analyed by Western blot, and (D) the quantitative analysis of SMP30 in normalization to β-actin was shown. Data represent means ± SEM (n=4). *****P<0.05, ******P<0.01 as compared with the control vehicle; $^{\#\#}P$<0.01 as compared with the testosterone (0.1 nM) treatment group; ^{+}P<0.01 as compared with the testosterone (10 nM) treatment group. The gels have been run under the same experimental conditions and cropped blots were shown. The entire of membrane pictures of Supplementary Fig. S1 were presented in the Supplementary Fig. S10. TE, testosterone; Flu, flutaminde; ns, non-specific binding.

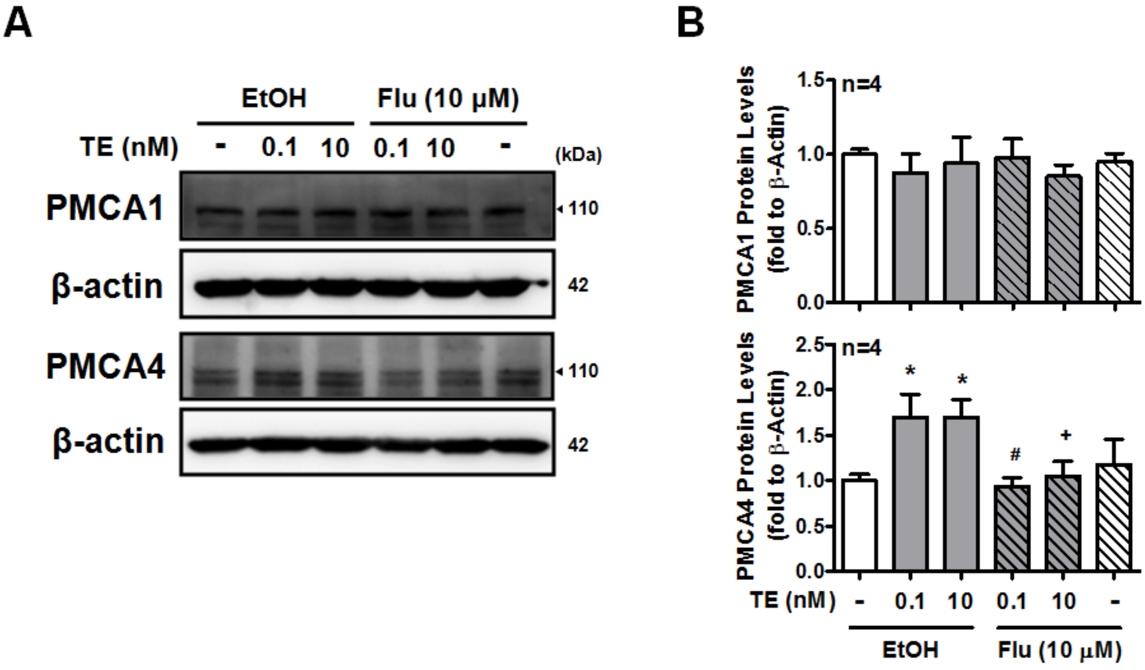

Supplementary Fig. S2. Effect of TE on the plasma membrane Ca^{2+} ATPase (PMCA) expression in NKR52E cells. (A) After resting for 12 h, NRK52E cells were pretreated with flutamind (10 μM) for 1 h and then treated with testosteone (0.1 and 10 nM), or EtOH as control vehicle, in serum- and phenol red-free DMEM medium for 12 h. At the end of time, cells were collected and the expression of PMCA isoforms, PMCA1 and PMCA4, protein levels were analyed by Western blot, and (B) the quantitative analysis of PMCA1 and PMCA4 in normalization to β-actin was shown, respectivety. Data represent means ± SEM (n=4). *****P<0.05 as compared with the control vehicle; $^{\#}P$<0.05 as compared with the testosterone (0.1 nM) treatment group; ^{+}P<0.01 as compared with the testosterone (10 nM) treatment group. The gels have been run under the same experimental conditions and cropped blots were shown. The entire of membrane pictures of Supplementary Fig. S2 were presented in the Supplementary Fig. S11. TE, testosterone; Flu, flutaminde.

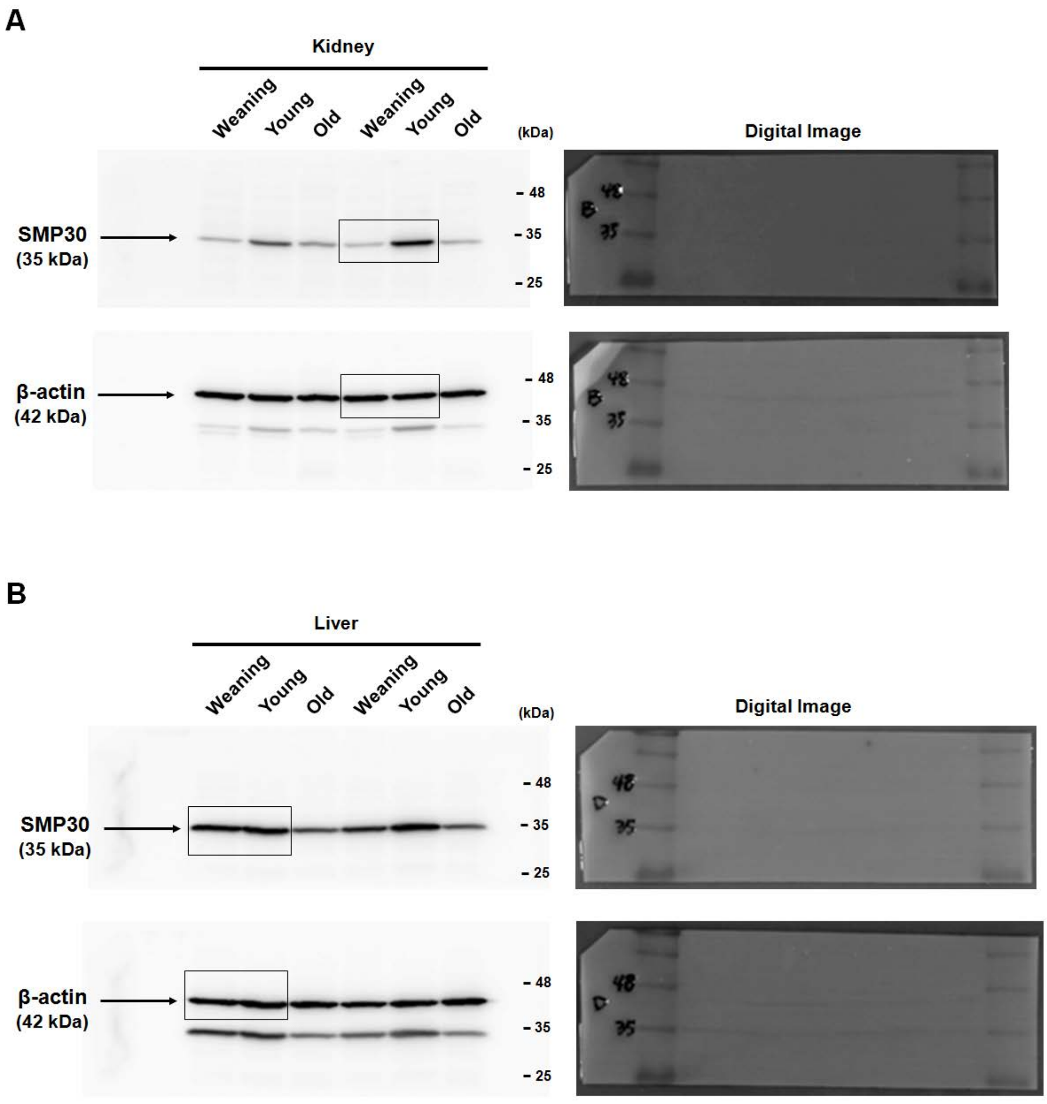

Supplementary Fig. S3. The original blot pictures of Fig. 1. in the text. Two individual rats of each group were shown in the membrane in the kidney and liver, respectively. (A) Corresponding to the section of kidney in Fig. 1B. (B) Corresponding to the section of liver in Fig. 1B. Size distribution of the molecular weight marker was shown in the digital images. The cropping lines and the molecular weight of target proteins were indicated.

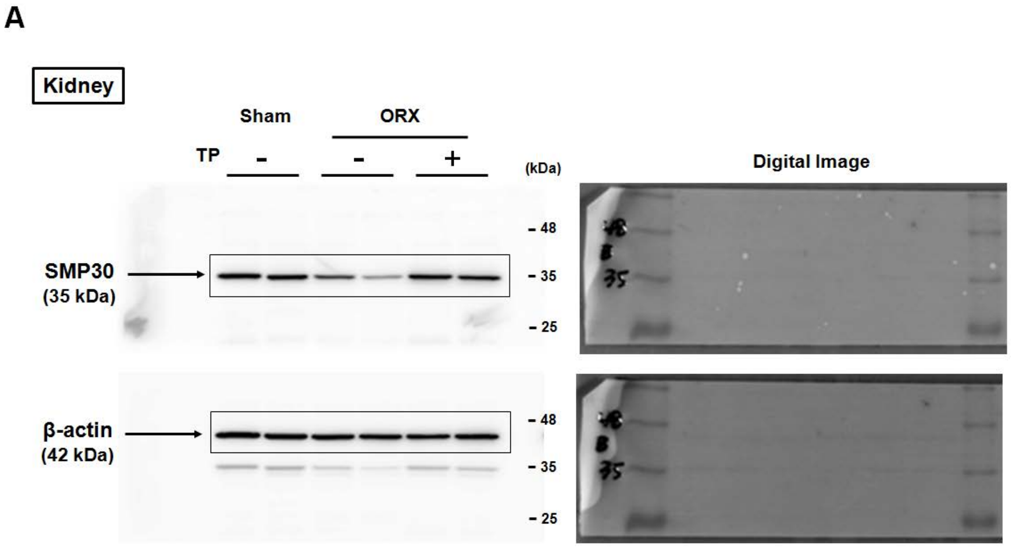

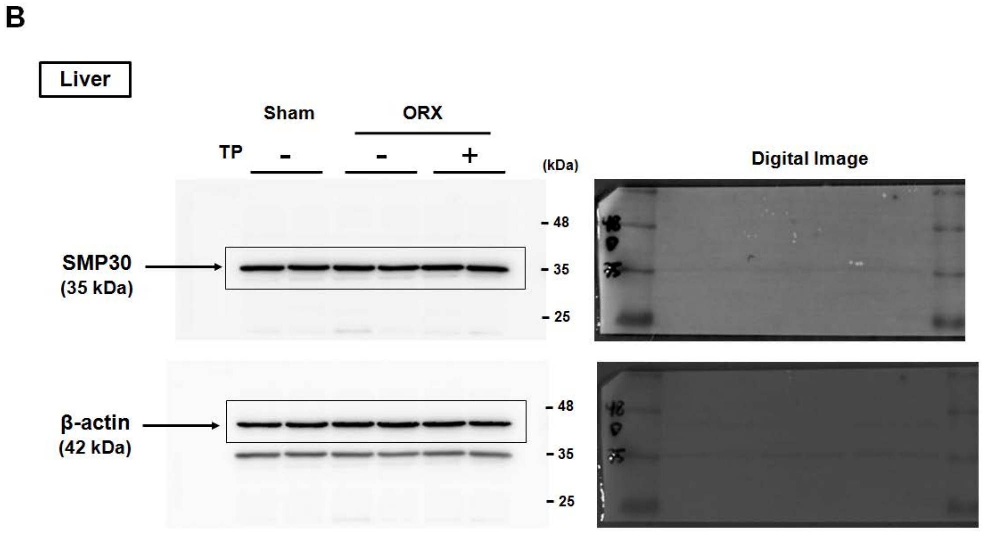

Supplementary Fig. S4. The original blot pictures of Fig. 2. in the text. Two individual rats of each group were shown in the membrane in the kidney and liver, respectively. (A) Corresponding to the section of kidney in Fig. 2A. (B) Corresponding to the section of liver in Fig. 2A. Size distribution of the molecular weight marker was shown in the digital images. The cropping lines and the molecular weight of target proteins were indicated.

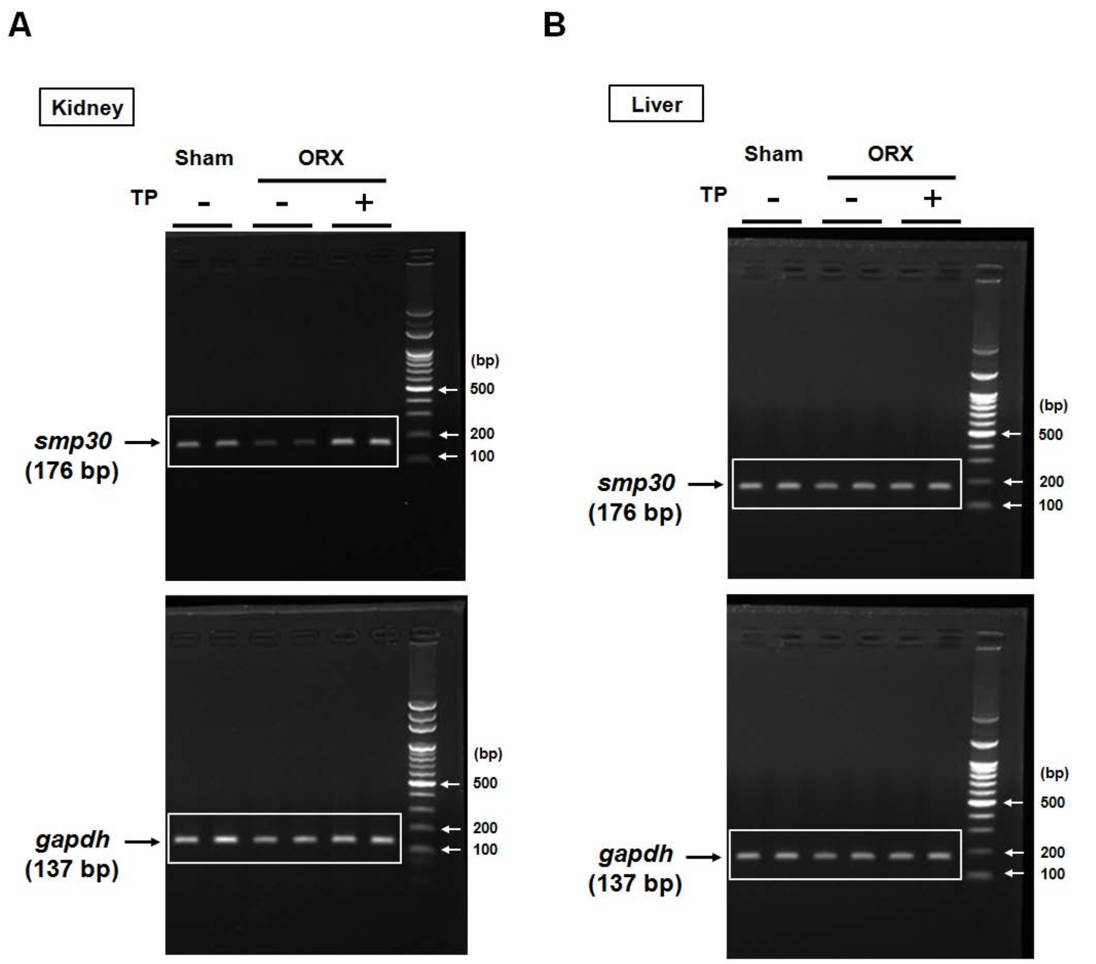

Supplementary Fig. S5. The original gel pictures of Fig. 2. in the text. Two individual rats of each group were shown in the membrane in the kidney and liver, respectively. (A) Corresponding to the section of kidney in Fig. 2D. (B) Corresponding to the section of liver in Fig. 2D. The cropping lines and the molecular weight of target PCR products were indicated.

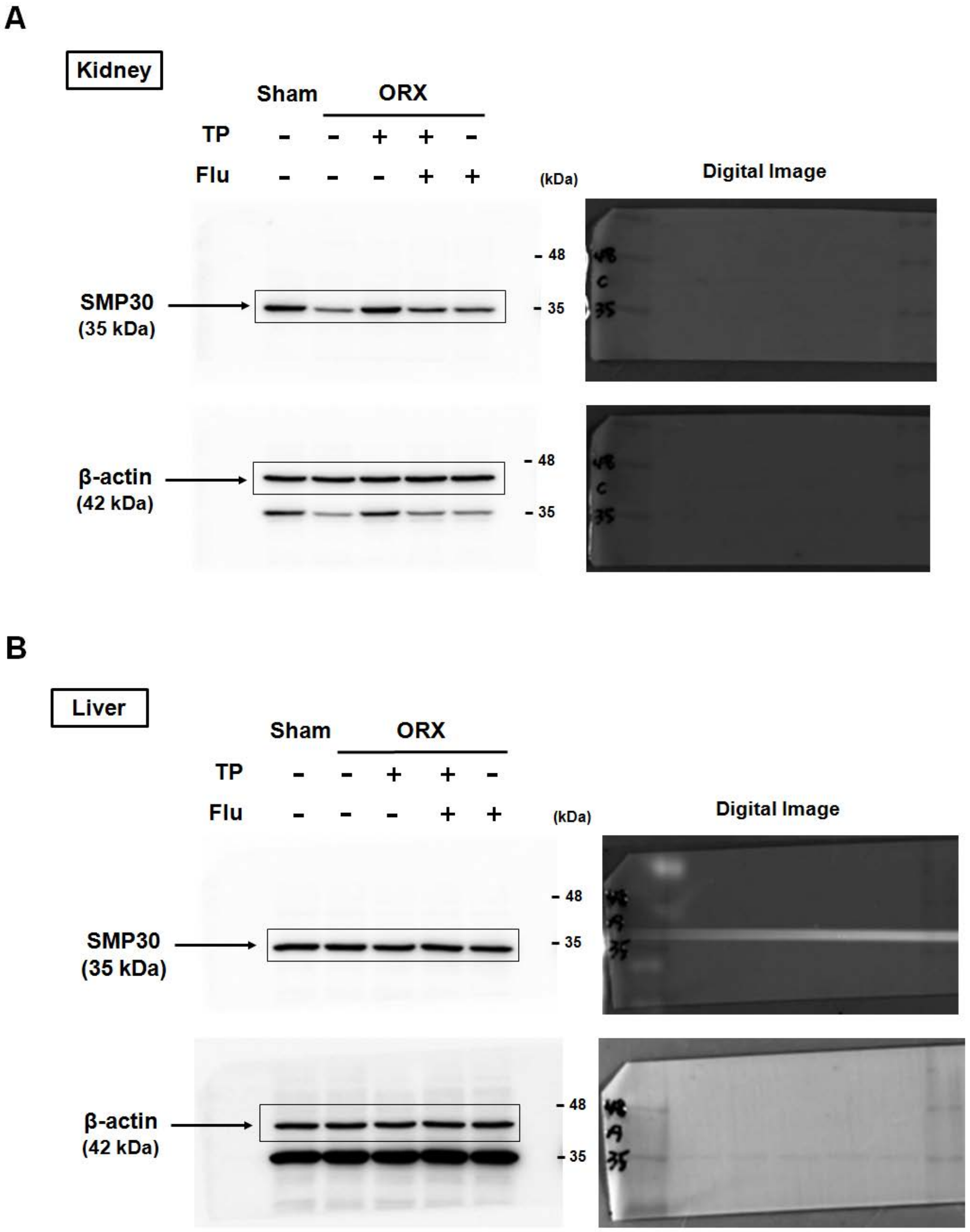

Supplementary Fig. S6. The original blot pictures of Fig. 3. in the text. (A) Corresponding to Fig. 3B. (B) Corresponding to Fig. 3C. Size distribution of the molecular weight marker was shown in the digital images. The cropping lines and the molecular weight of target proteins were indicated.

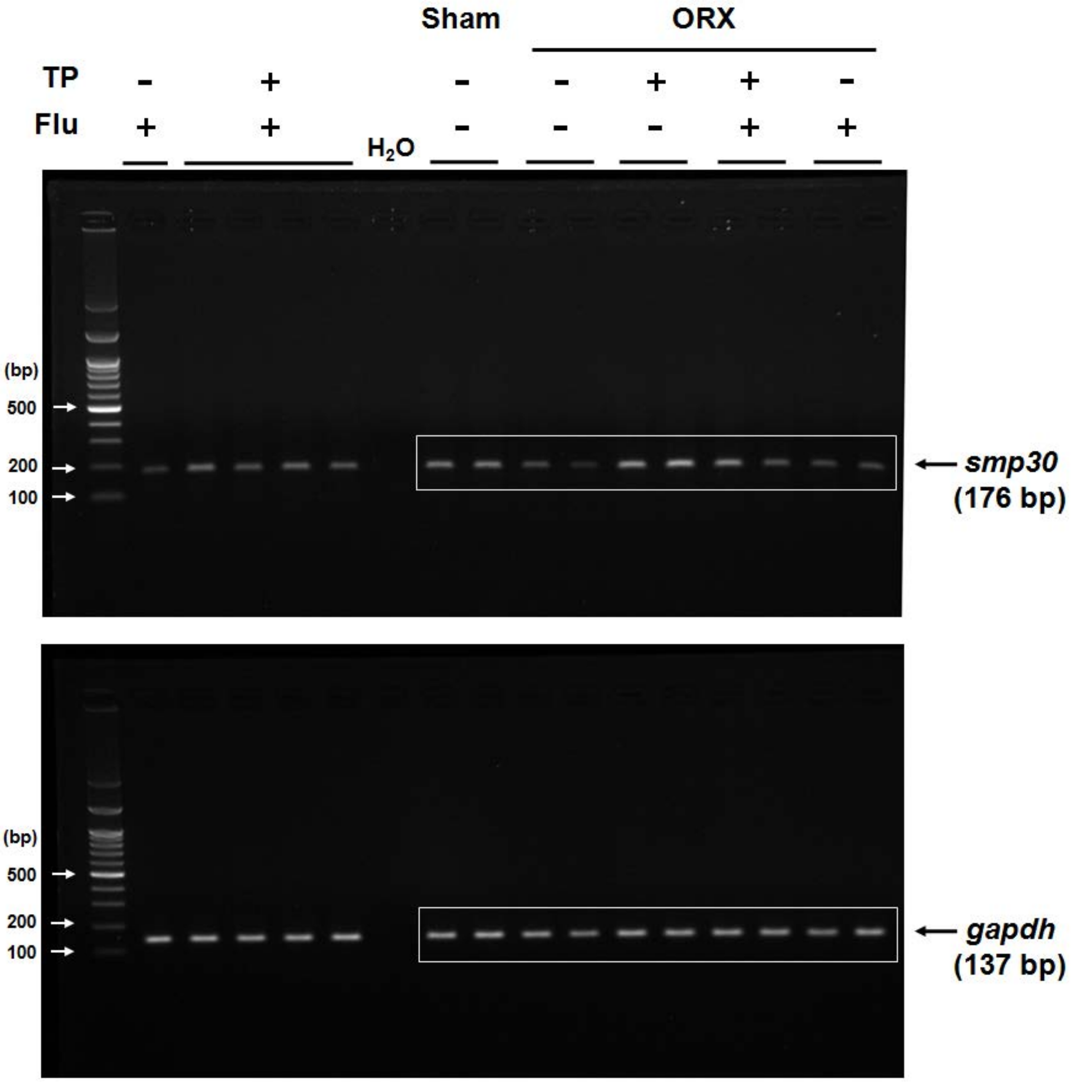

Supplementary Fig. S7. The original gel pictures of Fig. 3. in the text. Corresponding to Fig. 3D. Two individual rats of each group were shown in the gel. The cropping lines and the molecular weight of target PCR products were indicated. H_2O, as a negative control.

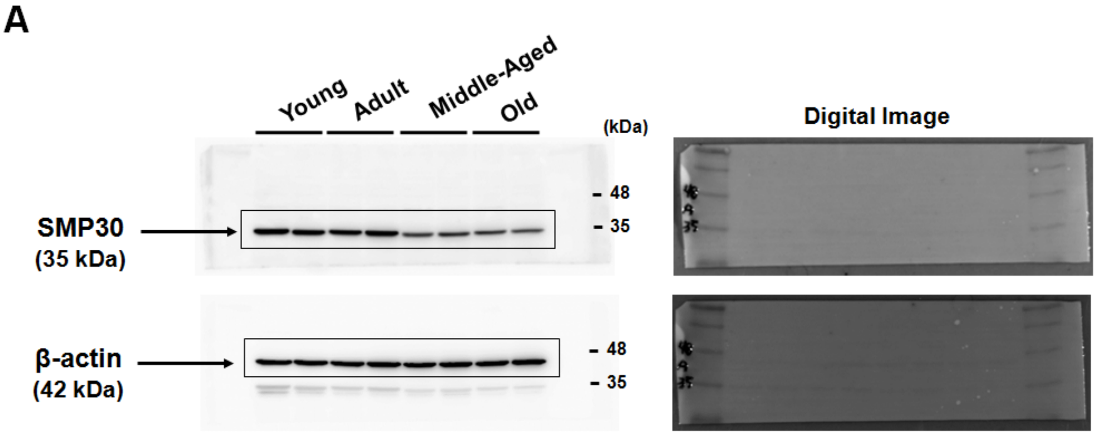

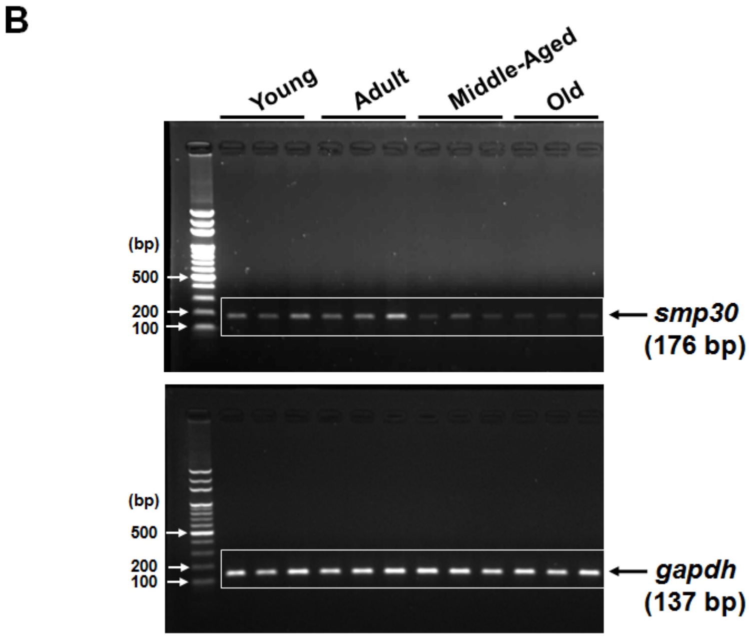

Supplementary Fig. S8. The original blot/gel pictures of Fig. 4. in the text. (A) Corresponding to Fig. 4A. Two individual rats of each group were shown in the membrane. Size distribution of the molecular weight marker was shown in the digital images. (B) Corresponding to Fig. 4B. Three individual rats of each group were shown in the gel. The cropping lines and the molecular weight of target proteins and PCR products were indicated.

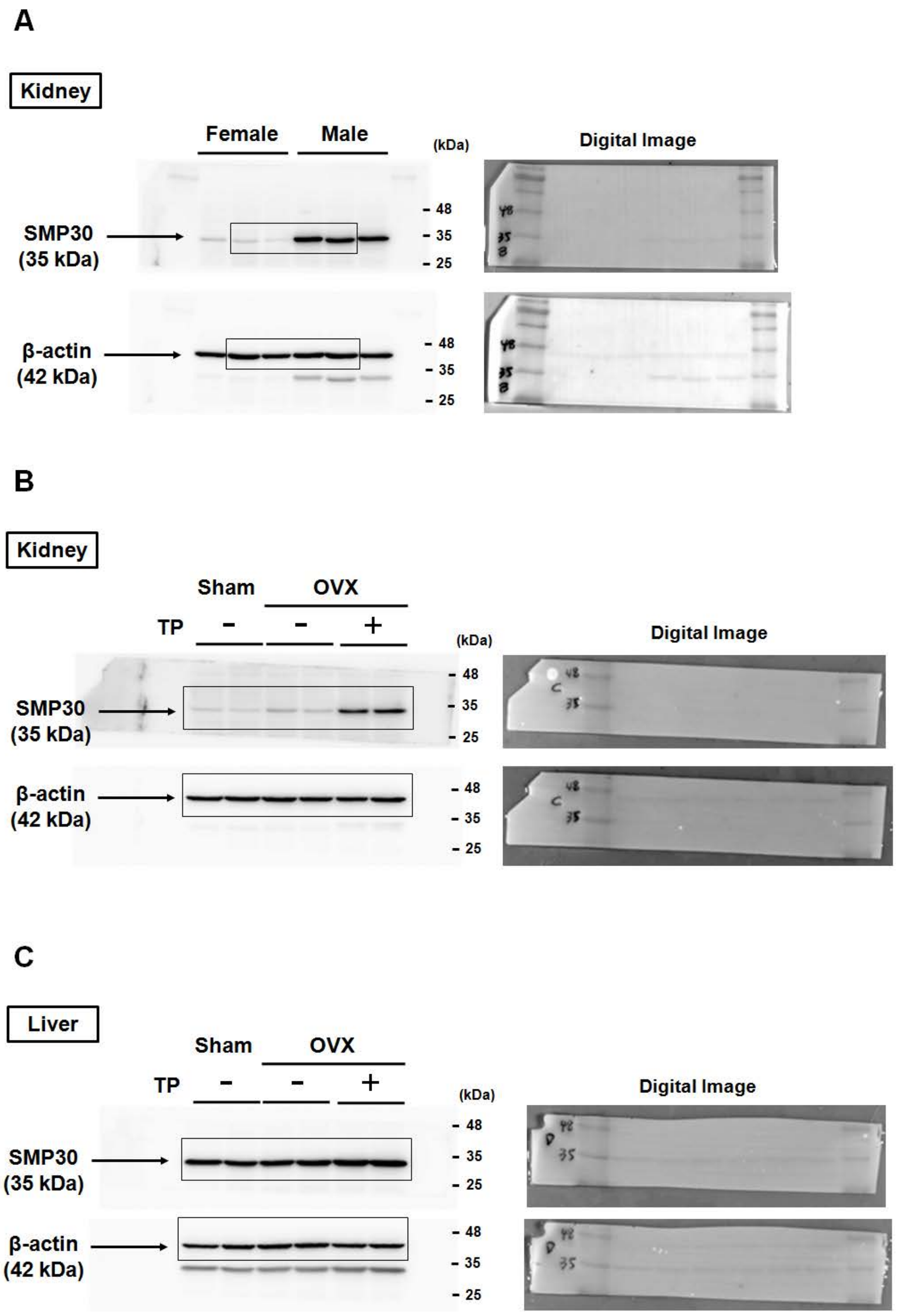

Supplementary Fig. S9. The original blot pictures of Fig. 5. in the text. (A) Corresponding to the section of kidney in Fig. 5A. Three individual rats of each group were shown in the membrane. (B) Corresponding to the section of kidney in Fig. 5B. (C) Corresponding to the section of liver in Fig. 5B. Two individual rats of each group were shown in the membrane in the kidney and liver, respectively. Size distribution of the molecular weight marker was shown in the digital images. The cropping lines and the molecular weight of target proteins were indicated.

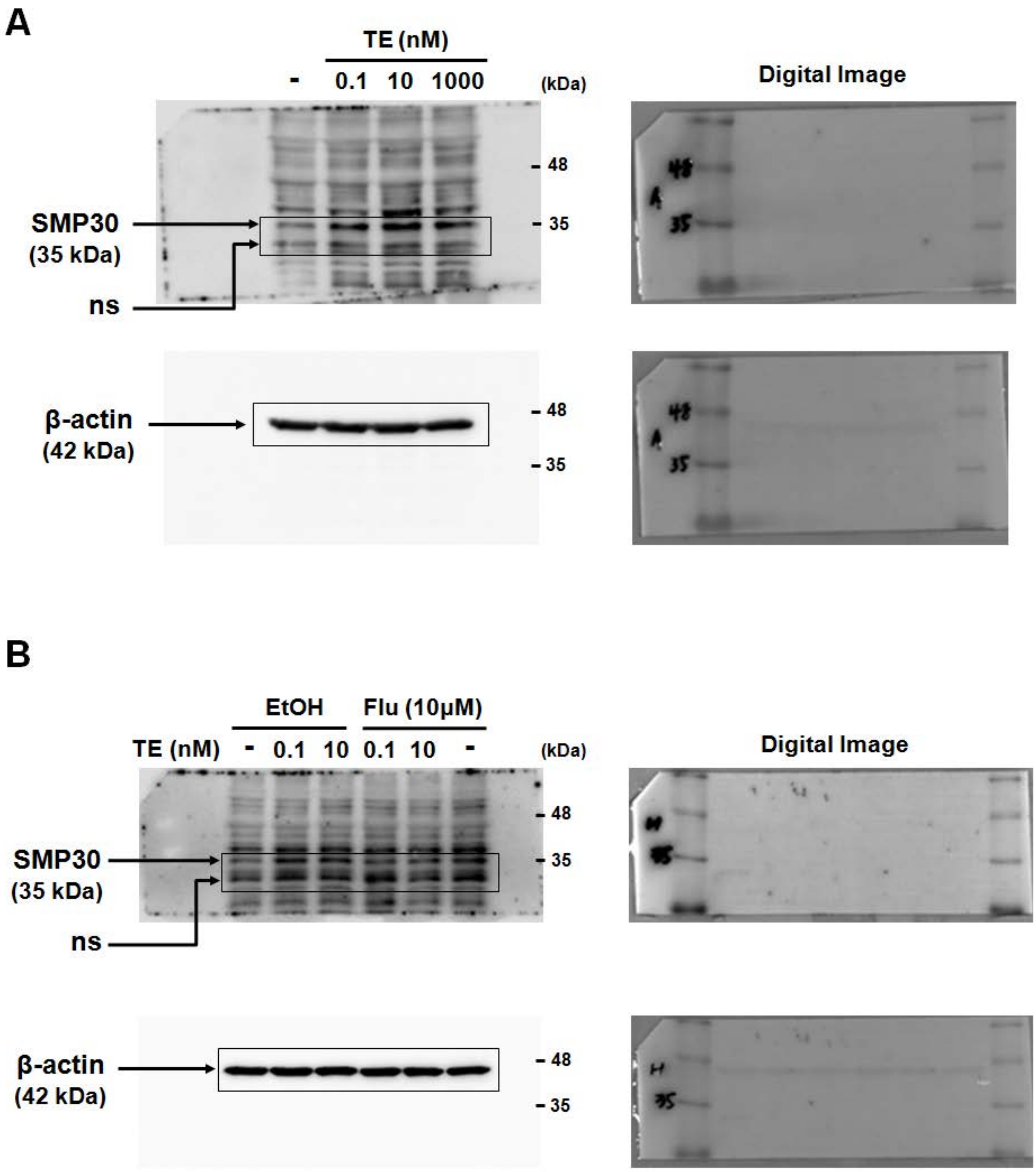

Supplementary Fig. S10. The original blot pictures of Supplementary Fig. S1. in the supplementary information. (A) Corresponding to Supplementary Fig. S1A. (B) Corresponding to Supplementary Fig. S1B. Size distribution of the molecular weight marker was shown in the digital images. The cropping lines and the molecular weight of target proteins were indicated. ns, non-specific binding.

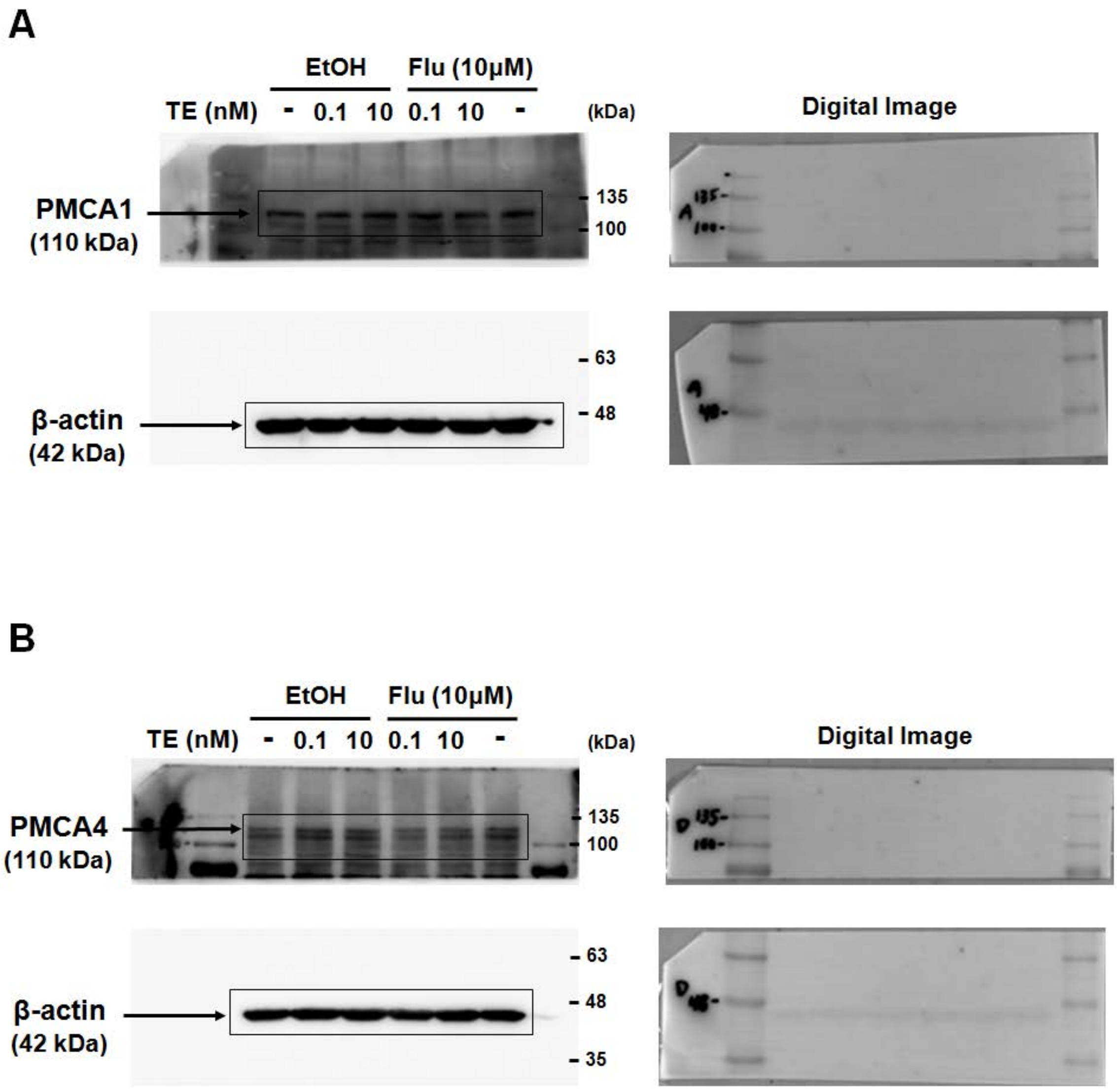

Supplementary Fig. S11. The original blot pictures of Supplementary Fig. S2. in the supplementary information. (A) Corresponding to the section of PMCA1 in the top panel of Supplementary Fig. S2A . (B) Corresponding to the section of PMCA4 in the bottom panel of Supplementary Fig. S2A .Size distribution of the molecular weight marker was shown in the digital images. The cropping lines and the molecular weight of target proteins were indicated.

Table S1. Effect of testosterone on plasma Ca^{2+}, plasma PTH, urinary volume, water and food intake levels

	#Rat number (n)	Sham	ORX	ORX+TP	ORX+TP+Flu	ORX+Flu	One-way ANOVA
Plasma Ca^{2+} (mg/dl)	5	9.7 ± 0.2	9.6 ± 0.3	9.5 ± 0.4	9.7 ± 0.5	10.3 ± 0.4	P = 0.367
Plasma PTH (ng/ml)	8	3.1 ± 0.3	3.4 ± 0.3	3.5 ± 0.2	3.0 ± 0.3	3.6 ± 0.3	P = 0.497
Urinary Volume (ml/24h)	6	23.0 ± 0.7	23.5 ± 2.0	22.3 ± 2.8	25.2 ± 1.2	23.3 ± 2.0	P = 0.825
Water Intake (ml/24h)	6	41.0 ± 2.3	42.8 ± 2.0	38.5 ± 3.4	42.7 ± 2.0	42.2 ± 4.3	P = 0.775
Food Intake (mg/24h)	6	22.7 ± 1.2	25.2 ± 1.6	21.5 ± 2.4	24.2 ± 0.9	23.6 ± 1.0	P = 0.399

Table S2. Aging effect on plasma Ca^{2+}, plasma PTH, urinary volume, water and food intake levels in male rats

	#Rat number (n)	Young	Adult	Middle-Aged	Old	One-way ANOVA
Plasma Ca^{2+} (mg/dl)	5	10.1 ± 0.3	-	10.1 ± 0.3	9.8 ± 0.2	P = 0.680
Plasma PTH (ng/ml)	8	3.9 ± 0.3	3.6 ± 0.2	4.0 ± 0.7	3.8 ± 0.4	P = 0.927
Urinary Volume (ml/24h)	5	24.2 ± 2.7	23.4 ± 3.0	24.0 ± 3.6	24.2 ± 3.2	P = 0.997
Water Intake (ml/24h)	5	44.2 ± 4.5	40.4 ± 6.2	40.8 ± 5.4	41.0 ± 6.9	P = 0.951
Food Intake (mg/dl)	5	21.5 ± 0.6	20.4 ± 1.2	21.4 ± 1.5	21.9 ± 2.0	P = 0.845
